# AF1q is a novel TCF7 co-factor which activates CD44 and promotes breast cancer metastasis

**DOI:** 10.18632/oncotarget.4136

**Published:** 2015-06-07

**Authors:** Jino Park, Michaela Schlederer, Martin Schreiber, Ryan Ice, Olaf Merkel, Martin Bilban, Sebastian Hofbauer, Soojin Kim, Joseph Addison, Jie Zou, Chunyan Ji, Silvia T. Bunting, Zhengqi Wang, Menachem Shoham, Gang Huang, Zsuzsanna Bago-Horvath, Laura F. Gibson, Yon Rojanasakul, Scot Remick, Alexey Ivanov, Elena Pugacheva, Kevin D. Bunting, Richard Moriggl, Lukas Kenner, William Tse

**Affiliations:** ^1^ James Graham Brown Cancer Center, Division of Blood and Bone Marrow Transplantation, Department of Medicine, University of Louisville School of Medicine, Louisville, KY, USA; ^2^ Ludwig Boltzmann Institute for Cancer Research, Vienna, Austria; ^3^ Comprehensive Cancer Center, Medical University of Vienna, Vienna, Austria; ^4^ Mary Babb Randolph Cancer Center, West Virginia University Health Science Center, Morgantown, WV, USA; ^5^ Department of Biochemistry, West Virginia University School of Medicine, Morgantown, WV, USA; ^6^ National Center for Tumor Diseases, German Cancer Research Center, Heidelberg, Germany; ^7^ Department of Laboratory Medicine, Medical University of Vienna and Core Facility Genomics, Core Facilities, Medical University of Vienna, Vienna, Austria; ^8^ Department of Hematology, Qilu Hospital, Shandong University School of Medicine, Jinan, Shandong, PR China; ^9^ Aflac Cancer and Blood Disorders Center of Children's Healthcare of Atlanta and Emory University School of Medicine, Atlanta, GA, USA; ^10^ Case Western University School of Medicine, Cleveland, OH, USA; ^11^ Cincinnati Children's Hospital Medical Center, Cincinnati, OH, USA; ^12^ Clinical Institute for Pathology, Medical University Vienna, Austria; ^13^ Department of Pharmaceutical Science, West Virginia University School of Medicine, Morgantown, WV, USA; ^14^ Institute of Animal Breeding and Genetics, University of Veterinary Medicine Vienna, Medical University of Vienna, Vienna, Austria; ^15^ Unit of Pathology of Laboratory Animals (UPLA), University of Veterinary Medicine, Vienna, Austria

**Keywords:** AF1q, TCF7, CD44, Wnt, metastasis

## Abstract

AF1q is an MLL fusion partner that was identified from acute myeloid leukemia (AML) patients with t (1; 11) (q21; q23) chromosomal abnormality. The function of AF1q is not yet fully known, however, elevated AF1q expression is associated with poor clinical outcomes in various malignancies. Here, we show that AF1q specifically binds to T-cell-factor-7 (TCF7) in the Wnt signaling pathway and results in transcriptional activation of CD44 as well as multiple downstream targets of the TCF7/LEF1. In addition, enhanced AF1q expression promotes breast cancer cell proliferation, migration, mammosphere formation, and chemo-resistance. In xenograft models, enforced AF1q expression in breast cancer cells also promotes liver metastasis and lung colonization. In a cohort of 63 breast cancer patients, higher percentages of AF1q-positive cancer cells in primary sites were associated with significantly poorer overall survival (OS), disease-free survival (DFS), and brain metastasis-free survival (b-MFS). Using paired primary/metastatic samples from the same patients, we demonstrate that AF1q-positive breast cancer cells become dynamically dominant in the metastatic sites compared to the primary sites. Our findings indicate that breast cancer cells with a hyperactive AF1q/TCF7/CD44 regulatory axis in the primary sites may represent “metastatic founder cells” which have invasive properties.

## INTRODUCTION

AF1q, a mixed-lineage leukemia (MLL) gene fusion partner, was discovered from acute myeloid leukemia (AML) patients whose leukemic blasts carry the t (1; 11) (q21; q23) chromosomal abnormality [1]. In normal hematopoietic tissues, AF1q expression is largely restricted to the differentiation of T-cells, but not to mature B- and T-cells (e.g., spleen and peripheral T-cells) [1]. MicroRNA29b (miR29b) binds to the AF1q 3′-untranslated region (3′-UTR) to inhibit its expression [2]. Although t (1; 11) (q21; q23) translocation is not frequently detected in various malignancies, elevated endogenous AF1q expression is commonly found in aggressive hematologic malignancies and many solid tumors (http://www.genecards.org/cgi-bin/carddisp.pl?gene=MLLT11) [3–5]. We have reported that elevated AF1q expression is associated with poor clinical outcomes in adult and pediatric acute myeloid leukemia as well as in myelodysplastic syndrome (MDS) [6–8]. In normal lymphopoiesis, AF1q is found to promote the emergence and migration of bone marrow pro-thymocytes to the thymus during T-cell development through interaction with the Notch signaling pathway [9]. However, the clinical significance of elevated AF1q expression in solid tumors has not been systematically explored. Laboratory studies by other groups suggest that AF1q may play a role in lung and breast cancer metastasis [10–12], which is the major cause of treatment failure and death in these malignancies [5, 10, 13]. In contrast to these observations, there are a few reports suggesting that AF1q has pro-apoptotic effects mediated by BAD or fenretinide-induced reactive oxygen species (ROS) in liver and ovarian cancer cell lines [3, 4, 14]. Most recently, a study further demonstrated that GATA3 suppresses breast cancer metastasis by inducing miR29b [15], which we have demonstrated to inhibit AF1q expression [2]. However, the underlying mechanisms of how AF1q is involved in promoting cancer metastasis are largely unclear. Thus, we investigated the underlying biological function of AF1q associated with breast cancer metastasis. Here, we demonstrate an important AF1q biological function for the first time as critical co-factor for TCF7 binding to the CD44 promoter, resulting in transcriptional activation of CD44. This is an important mechanism related to breast cancer cell growth, therapeutic resistance, distant metastasis, and clinical outcomes. Furthermore, we validate our *in vitro* and *in vivo* observations in a cohort of breast cancer patients and demonstrate that elevated AF1q expression is significantly associated with poorer survival and a higher incidence of distant (brain) metastasis.

## RESULTS

### AF1q expression between breast normal epithelial and cancer cell lines as well as breast normal and cancer tissues

**Figure 1:**
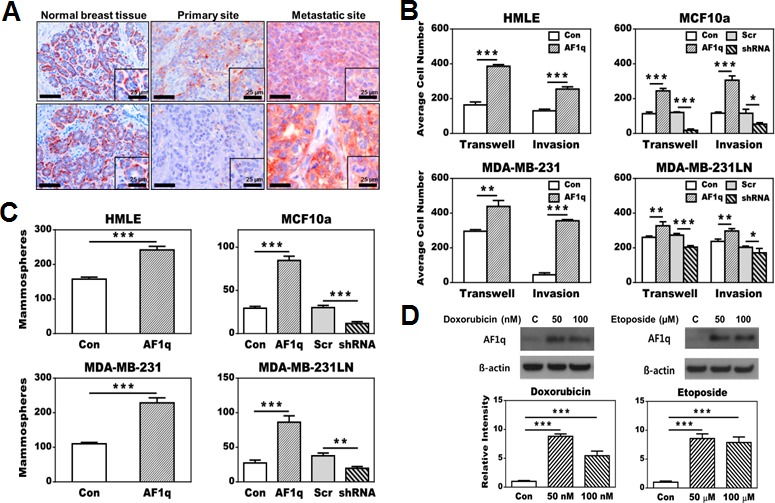
Effects of AF1q expression in breast normal epithelial (HMLE and MCF10a) and cancer cell lines (MDA-MB-231 and MDA-MB-231LN) **A.** Immunohistochemistry (IHC) staining shows a higher frequency and intensity of AF1q-positive cancer cells in metastatic sites compared to primary sites. In normal breast tissue, AF1q staining was restricted to myoepithelial cells rather than ductal/glandular epithelial cells. Scale bars = 50 μm. **B.** Enforced AF1q expression in breast normal epithelial and cancer cell lines promoted transwell migration and matrigel invasion compared to their correspondent empty vector (EV) controls. Using short hairpin RNA (shRNA) to suppress the endogenous AF1q expression in MCF10a and MDA-MB-231LN inhibited transwell migration and matrigel invasion compared to their correspondent scrambled RNA (Scr) controls. **C.** Enforced AF1q expression promoted mammosphere formation in breast normal epithelial cell lines (HMLE and MCF10a) and tumorsphere formation in breast cancer cell lines (MDA-MB-231 and MDA-MB-231LN); suppression of the endogenous AF1q expression with shRNA in MCF10a or MDA-MB-231LN inhibited mammosphere or tumorsphere formation, respectively. **D.** Endogenous AF1q expression in MDA-MB-231LN was significantly elevated after treatment for 24 hours with two different concentrations of doxorubicin or etoposide compared to its controls. Cell viability was significantly decreased with 100 nM doxorubicin and 100 uM etoposide (lower than 50%), but not with lower concentration of both. Data are mean values ± s.d. of three independent experiments. *P* values were calculated using student *t* test (*, *P* < 0.05; **, *P* < 0.01; ***, *P* < 0.001).

We examined AF1q expression in breast normal epithelial and cancer cell lines and detected AF1q in one of two immortalized breast normal epithelial cell lines (MCF10a) and in three of eight cancer cell lines (MDA-MB-231LN, MDA-MB-435, and Hs578T) (Supplementary Figure S1A). In contrast to a report by Chang et al [12], we could not detect AF1q expression in MDA-MB-231 cells. This discrepancy possibly results from different antibodies used in the experiments. We used a commercially co-developed high affinity rabbit monoclonal anti-AF1q antibody, whereas Chang et al. used a mouse anti-AF1q antibody. As expected, AF1q expression was detected in most of the breast normal epithelial and cancer cell lines, except ZR75-1, where it was also inversely correlated with miR29b expression (Supplementary Figure S1B). The prevalence of AF1q and miR29b expression in tested breast cancer cell lines was similar to prior observations in AML patients [2] supporting the notion that miR29b probably also inhibits AF1q expression in most breast cancer cells. Immunohistochemistry (IHC) staining of breast normal tissue revealed that AF1q expression was largely restricted to progenitor-associated myoepithelial cells and was not observed in ductal/glandular epithelial cells (Figure 1A). However, AF1q expression became prominently positive in cancerous ductal/glandular epithelial cell foci. In contrast to the significantly higher prevalence of AF1q-positive cancer cells in metastatic sites, we found that AF1q-positive cancer cells were much less frequent in primary sites of breast cancer (Figure 1A). This observation suggests that AF1q positive breast cancer cells may have outgrowth and migration advantages with current treatment modalities, which we further tested in the following *in vitro* system.

### Enforced AF1q expression resulted in oncogenic growth, migration, invasion, and drug resistance in breast normal epithelial and cancer cell lines

To investigate the biological characteristics of AF1q, we first experimentally enforced or suppressed AF1q expression in two breast normal epithelial cell lines, MCF10a and HMLE, and two breast cancer cell lines, MDA-MB-231LN (invasive sub line from MDA-MB-231) and MDA-MB-231. Of these, one of the breast normal epithelial cell line MCF10a and one of the invasive breast cancer cell line MDA-MB-231LN had endogenous AF1q expression, whereas the other breast normal epithelial cell line HMLE and the less invasive breast cancer cell line MDA-MB-231 had undetectable AF1q expression (Supplementary Figure S1A). We then used a lentiviral transduction system to enforce AF1q expression in these 4 cell lines and to suppress AF1q expression in cell lines with endogenous AF1q expression (MCF10a and MDA-MB-231LN). As expected, enforced AF1q expression in all cell lines, regardless of their endogenous AF1q expression status, consistently promoted cell growth, trans-well migration, invasion, mammosphere formation, and chemotherapy resistance compared to their control parental cell lines with empty vectors (Figure 1B–1C; Supplementary Figure S2A–C). Suppressed AF1q expression by shRNA (shown here as the representative shRNA knockdown out of three different sets of shRNA tested) in MCF10a and MDA-MB-231LN could effectively attenuate those oncogenic characteristics compared to scrambled controls (Figure 1B–1C; Supplementary Figure S2A–C). Treatment of breast cancer cells MDA-MB-231LN with doxorubicin or etoposide, chemo-agents commonly used for treating breast cancer, resulted in a further activation of the endogenous AF1q expression (Figure 1D). In addition, enforced AF1q expression in breast normal epithelial HMLE cells produced a side population of cells that acquired anoikis-resistance, a feature only seen in pro-metastatic and aggressive cancer cells (Supplementary Figure S2D). Together, these observations suggest that elevated AF1q expression is highly associated with aggressive growth, and an invasive and metastatic phenotype. Given that breast cancer chemotherapy agents further activated endogenous AF1q expression in MDA-MB-231LN and potentially promoted a more aggressive phenotype after therapeutic selection, we hypothesized that AF1q-positive breast cancer cells in the primary site might represent “metastatic founder cells”. They could effectively resist chemotherapy and “escape” from the primary site resulting in migration to distant organs. Thus, we next tested this hypothesis in xenograft models.

### AF1q interacts with TCF7 and enhances the Wnt signaling pathway

**Figure 2:**
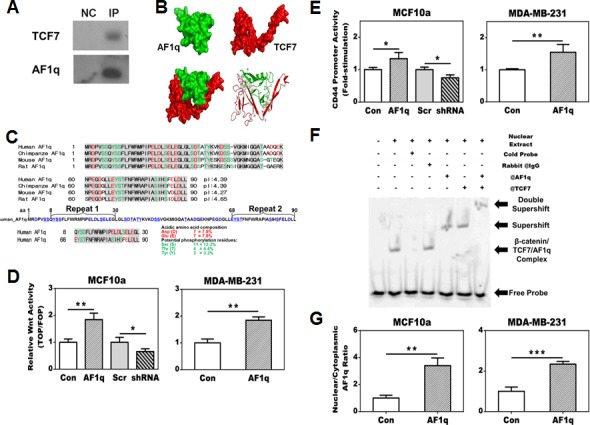
AF1q is a cofactor of TCF7 and forms a transcriptional complex with TCF7, LEF1 and β-catenin which leads to a transcriptional activation of CD44, one of the downstream targets of the Wnt signaling pathway **A.** AF1q and TCF7 interaction was confirmed in MCF10a by co-immunoprecipitation of TCF7 with anti-AF1q antibody. NC is negative control for nonspecific binding. **B.** The Schrodinger program generates a three-dimensional AF1q and TCF7 interaction model based on yeast 2-Hybrid results; TCF7 forms a glove-like shape to envelope the entire AF1q protein. **C.** The AF1q sequence is highly conserved across mammalian species, in particular the acidic (E and D in red) and phosphorylatable amino acids (S, T and Y in green) are highlighted. The acidic nature is best reflected by the low isoelectric point (pI) of the peptides across different species. There is a repeated internal protein motif region (aa 8-30 and aa 68-90) in the 9 kDa peptide of AF1q shown below the AF1q amino acid sequences between different mammalian species. **D.** TOP/FLOP flash reporter assays show that enforced AF1q expression enhanced the Wnt signaling activity; the suppressed endogenous AF1q expression by shRNA attenuated the Wnt signaling activity. **E.** AF1q enhanced CD44 promoter activity, as shown by comparing transfection of the pGL4-CD44 promoter with the pGL4-EV reporter control. **F.** Super-shift assay using the MCF10a nuclear extracts showed that AF1q and TCF7 directly bind to the CD44 promoter. **G.** The AF1q nuclear shift after enforced AF1q expression was confirmed in fractionated cytoplasm and nuclear extracts from MCF10a cells. Data are mean values ± s.d. of three independent experiments. *P* values were calculated using student *t* test (*, *P* < 0.05; **, *P* < 0.01; ***, *P* < 0.001).

To determine the biochemical function of AF1q with a size of only 9 kDa, we sought to identify its potential interactive partners. Yeast-2-Hybrid (Y-2-H) screens suggested that AF1q may bind to TCF7 (also known as TCF1, overlapped with 8 hits) and LEF1 (overlapped with 2 hits) (Supplementary Table S1; Supplementary Figure S3A), both of which are transcription factors with high mobility group (HMG) boxes [16, 17]. Immunoprecipitation (IP) experiments using the newly generated AF1q rabbit monoclonal antibody confirmed an interaction between AF1q and TCF7 (Figure 2A), but not between AF1q and LEF1. Our explanation is that AF1q/LEF1 interaction may be weak or indirect because a small 9 kDa protein has little probability to simultaneously bind to two relatively large size proteins. Moreover, TCF7 is known to bind to LEF1 that may result in a false Y-2-H positive result of AF1q and LEF1 interaction. This notion is also supported by the finding that only two interactive hits were demonstrated between AF1q and LEF1, but eight interactive hits were observed between AF1q and TCF7 (Supplementary Table S1). A protein-protein interaction model, generated based on the AF1q and TCF7 interactive amino acid sequences suggested by Y-2-H, suggests that TCF7 forms a glove-like shape to envelope the entire AF1q protein (Figure 2B).

Structurally, AF1q has highly acidic peptide regions that are highly conserved between human, chimpanzees, or rodent species that fulfill the criteria of an acidic blob, a typical feature for cofactors (Figure 2C). AF1q contains 14 acidic amino acids (D and E highlighted in red), which results in a low isoelectric point of 4.53. Furthermore, it could become more acidic if potential phosphorylation residues (18 S, T, and Y amino acids, highlighted in green) were further phosphorylated. Thus, we suggest that AF1q is a previously unrecognized co-factor associated with the TCF7/LEF1/β-catenin transcriptional complex. We also noted an internal peptide repeat (Figure 2C) located at both ends of the AF1q protein that was indicative of similar binding interfaces.

To further examine whether AF1q can change the Wnt signaling activity, we utilized the TOP/FOP flash reporter assay in breast normal epithelial cells MCF10a and cancer cells MDA-MB-231 with enforced/suppressed AF1q expression. MDA-MB-231LN was transformed to constantly express luciferase, so we chose MDA-MB-231 instead of MDA-MB-231LN. We observed that Wnt signaling activity was significantly enhanced after enforced AF1q expression in both cell lines, but significantly reduced in MCF10a cells after suppression of endogenous AF1q expression (Figure 2D). Although the enhancement or reduction of the relative Wnt activity after enforced or suppressed AF1q expression was not as dramatic as usually seen with other transcription factors in TOP/FOP assays, these findings are consistent with a co-factor function for AF1q rather than a transcription factor in the Wnt signaling pathway. Next, we used a reporter assay of a luciferase promoter of CD44, one of Wnt signaling pathway's downstream targets which was associated with breast cancer stem cell metastasis and AML stem cell homing [18–20]. Indeed, enforced AF1q expression could activate the CD44 promoter (Figure 2E). Super-shift and electrophoretic mobility shift assay (EMSA) further confirmed that AF1q directly interacted with TCF7 and enhanced the binding affinity of the complex (Figure 2F; Supplementary Figure S3B). Taken together, the data from Figure 2A and 2F confirm AF1q and TCF7 binding in two different experimental settings. In parental MCF10a cells, AF1q expression is detected in both nuclei and cytoplasm. However, enforced AF1q expression in MCF10a and MDA-MB-231 cells resulted in activation of the Wnt signaling pathway with significantly more AF1q protein shifted into the nuclear compartment compared to its control cells (Figure 2G). These results indicate that AF1q functions as a co-factor within the TCF7/LEF1/β-catenin transcriptional complex.

### Activation of CD44 is essential for the AF1q oncogenic function

**Figure 3:**
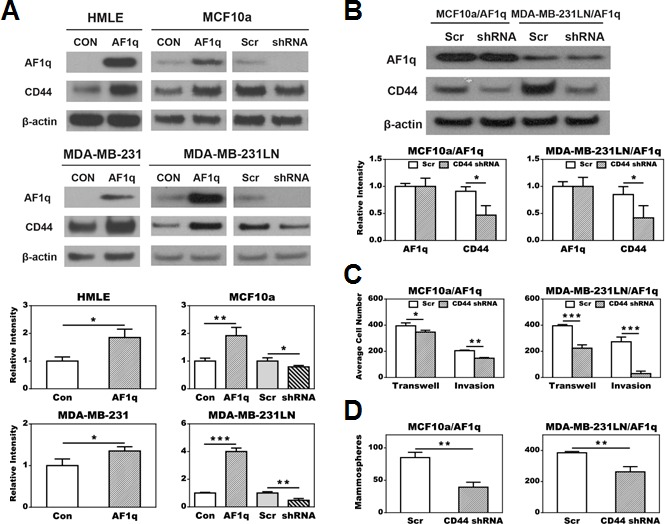
AF1q mediated cell transwell migration, matrigel invasion, and mammosphere (tumor sphere) formation essentially depends on activation of CD44 **A.** CD44 expression was up-regulated after enforced AF1q expression in all tested cell lines. CD44 expression was attenuated after suppressed endogenous AF1q expression by shRNA in MCF10a and MDA-MB-231LN. **B.** Suppressed CD44 expression by shRNA in MCF10a/AF1q and MDA-MB-231LN/AF1q did not affect AF1q expression. **C.** Suppressed CD44 expression by shRNA in MCF10a/AF1q and MDA-MB-231LN/AF1q attenuated AF1q-mediated transwell migration and matrigel invasion. **D.** Suppressed CD44 expression by shRNA in MCF10a/AF1q and MDA-MB-231LN/AF1q attenuated AF1q-mediated mammosphere/tumor sphere formation. Data are mean values ± s.d. of three independent experiments. *P* values were calculated using student *t* test (*, *P* < 0.05; **, *P* < 0.01; ***, *P* < 0.001).

Enforced AF1q expression significantly increased CD44 protein expression in all tested cell lines. Conversely, suppression of endogenous AF1q in MCF10a and MDA-MB-231LN cells resulted in decreased CD44 protein expression (Figure 3A). Because CD44 is associated with breast cancer cell invasion [21], we want to determine whether activation of CD44 is essential for the oncogenic and invasive properties of AF1q. Hence we chose to knock down CD44 expression in MCF10a/AF1q and MDA-MB-231LN/AF1q cells without alteration of enforced AF1q expression (Figure 3B). Cell migration, invasion, and mammosphere formation induced by AF1q were all effectively reversed after CD44 knockdown (Figure 3C–3D). Similarly, IHC staining of AF1q or CD44 in human breast normal and tumor specimens showed that AF1q expression areas roughly matched with the CD44 signals (Supplementary Figure S4). These results suggest that the AF1q oncogenic and invasive properties are directly associated with CD44 activation.

### The oncogenic and invasive properties associated with AF1q overexpression in breast cancer cells are orchestrated through enhancing and attenuating activity in a group of downstream targets of the Wnt signaling pathway

**Figure 4:**
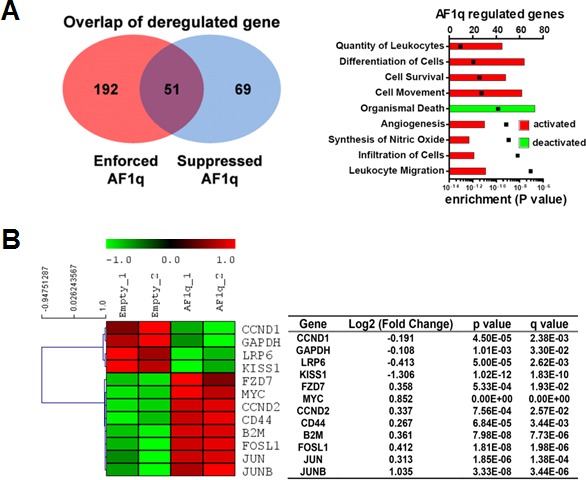
AF1q is tightly associated with a metastasis gene cluster **A.** RNA-seq analysis suggests that enforced or suppressed AF1q expression by lentiviral system led to up-regulation or down-regulation of 243 and 120 genes, respectively. Pathway activities significantly affected by enforced AF1q expression are illustrated in bar graph (www.ingenuity.com; black squares indicate *P*-value). **B.** Hierarchical clustering of AF1q associated multiple downstream targets in the Wnt signaling pathway are depicted in a Heat map. The dendrogram groups genes into distinct groups according to their distance from each other. The distance measure is shown on the horizontal axis. Results are from MDA-MB-231LN with enforced AF1q expression. Down-regulated genes are shown in green and up-regulated genes in red. Fold change corresponds to AF1q over Ctrl-transduction. The table shows FDR-adjusted *p*-value (‘*q*-value) as well as the uncorrected *p*-value of the test statistic.

RNA sequencing (RNA-seq) of MDA-MB-231LN cells revealed significant changes in expression of 210 genes after enforced or suppressed AF1q expression (*p* < 0.05). Among them, the expression pattern of 51 genes was increased by enforced and decreased by suppressed AF1q expression (Figure 4A, left panel; Supplementary Table S2). These genes could be clustered into pathways that are strongly associated with cell migration, invasion and survival (Figure 4A, right panel; Supplementary Table S3), aligned with the assertion that AF1q is a critical gene related to aggressive/invasive behavior of breast cancer as shown in Figures 1–2. In addition to CD44 activation, we examined the expression status of multiple Wnt downstream targets from RNA-seq and found that the expression of other Wnt targets was collectively orchestrated into two groups: (i) Significantly up-regulated genes from one group were associated with stem cell self-renewal (Myc) [22], Wnt signaling receptor that can down-regulate tumor suppressive APC gene (FZD7) [23], stemness maintenance (CCND2) [24], and AP-1 family transcription factors associated with tumor growth (Fosl1 and Jun) [25]. (ii) In contrast, significantly down-regulated genes from another group were associated with tumor metastasis suppression (KISS1) [26–30], retinoblastoma (Rb) gene positive regulator (CCND1) [31], and a canonical Wnt antagonist (LRP6 through DDK1) [32] (Figure 4B). Collectively, AF1q effectively participated in regulation of Wnt signaling target genes that are important for breast cancer growth, differentiation, and metastasis.

### Enforced AF1q expression promotes breast cancer cell growth and distant organ colonization/metastasis in the NOD/SCID mouse models

**Figure 5:**
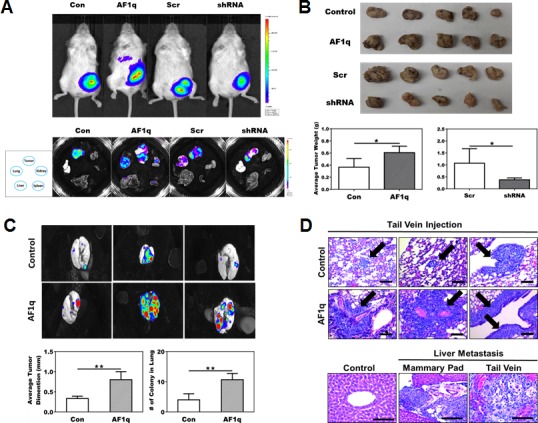
NOD/SCID xenograft mouse models confirm the association of AF1q with breast cancer metastasis **A.** Representative image of mice injected with MDA-MB-231LN Con, AF1q, Scr and shRNA cells (upper panel) and tumor, liver, lung, kidney and spleen tissues dissected from the 4 treated groups of mice (lower panel) at 4 weeks after injection in mammary fat pad model. **B.** Tumors were harvested from mammary fat pad injection model and size and weight were measured at 4 weeks after injection. MDA-MB-231LN/AF1q injected mice grew significantly larger tumor masses compared to MDA-MB-231LN/EV control mice. Suppressed the endogenous AF1q expression with shRNA in MDA-MB-231LN were significantly smaller and lighter tumors. The tumor weight is shown as the mean ± s.d. *P* values were calculated using student *t* test (*, *P* < 0.05; **, *P* < 0.01; ***, *P* < 0.001). **C.** The tail vein injection model, MDA-MB-231LN/AF1q significantly grew larger tumor masses than MDA-MB-231LN/EV. Data were mean values ± s.d. *P* values were calculated using student *t* test (*, *P* < 0.05; **, *P* < 0.01; ***, *P* < 0.001). **D.** Representative histology sections from the tail vein injection model MDA-MB-231LN/AF1q displayed more aggressive histologic features than controls (upper panel); arrows indicate areas of metastasis formation. Obvious liver metastases developed in both mammary pad (2/3 mice) and tail vein injection (1/3 mice) models (lower panel), but not in controls. Scale bars = 50 μm.

In an attempt to examine the *in vivo* oncogenic effect of AF1q, we evaluated MDA-MB-231LN/AF1q growth, colonization, and metastasis in two types of NOD/SCID mouse models by using mammary fat pad implantation and tail vein injection. In mammary fat pad implantation model, MDA-MB-231LN/AF1q demonstrated significantly aggressive local growth and metastasis capacity compared to the MDA-MB-231LN/Empty Vector (EV) control. In contrast, MDA-MB-231LN/shRNA demonstrated effectively attenuated local growth and metastasis compared to the MDA-MB-231LN/Scr control (Figure 5A–5B). In a tail vein injection model, MDA-MB-231LN/AF1q demonstrated significantly increased lung colonization capacity compared to the MDA-MB-231LN/EV control (Figure 5C). Most strikingly, early liver metastases developed within 4-weeks in both mammary fat pad implantation and tail vein injection models with MDA-MB-231LN/AF1q cells (Figure 5D lower panel). However, none of the MDA-MB-231LN/EV control mice in both models developed any organ metastasis during the same experimental period. The local and metastatic foci formed by MDA-MB-231LN/AF1q in both models generally showed more aggressive growth patterns reflected by larger size of tumor cells and polymorphic nuclei, an increased nuclear to cytoplasmic ratio, and increased atypical mitoses compared to its EV controls (Figure 5D upper panel). These observations strongly suggest that breast cancer cells with hyperactive AF1q may be able to supersede into distant organs and at a later time point overcome the blood-organ barrier.

### High percentage of AF1q-positive cancer cells in primary breast cancer sites is associated with poor overall survival (OS), disease-free survival (DFS) and brain metastasis-free survival (b-MFS)

**Figure 6:**
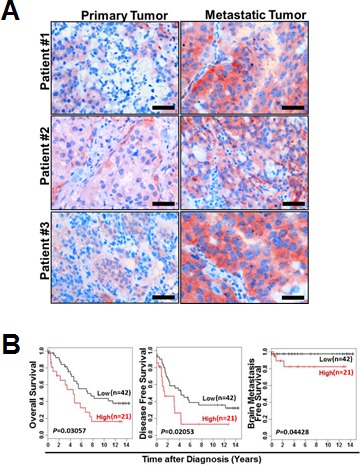
AF1q expression in breast cancer is positively associated with metastasis **A.** Representative sections of paired primary/metastatic tissue from 3 breast cancer patients demonstrated a significantly higher frequency and intensity of AF1q-positive breast cancer cells in metastatic sites (lymph nodes and lung) than in primary sites. Scale bars = 50 μm. **B.** Breast cancer patients with ≥50% AF1q-positive cancer cells in primary diagnostic sites (high, *n* = 21) had significantly poorer overall survival (OS), disease-free survival (DFS) and brain metastasis-free survival (b-MFS) than patients with < 50% AF1q-positive cancer cells (Low, *n* = 42).

We validated our *in vitro* and *in vivo* observations in breast cancer tissue microarrays (TMAs) from a cohort of 63 breast cancer patients. Among them, 3 patients with both primary and metastatic lesions were available for analysis. These paired primary and metastatic lesions from the same patients had unique biological and clinical value to study metastatic mechanisms in breast cancer patients that mouse models were unable to fully accomplish. These materials could provide direct and dynamic evidence of the AF1q-positive tumor cell population changes between primary and metastatic lesions within the same patient. Importantly, we found that the frequency and intensity of AF1q-positive tumor cells was significantly higher in metastatic sites (AF1q-positive cells metastasize into lymph nodes and lung) than in primary sites (Figure 6A). When we analyzed the AF1q expression in the primary site of the entire patient cohort, we found that a higher fraction (≥50%) of AF1q-positive cells in the primary site was associated with significantly poorer OS, DFS, and b-MFS (Figure 6B). In addition, estrogen receptor negativity (*p* = 0.006) and lymph node involvement (*p* = 0.028) were associated with high AF1q expression levels (Supplementary Table S4). Taken together, these data strongly support the notion that AF1q is a poor prognostic marker in breast cancer.

## DISCUSSION

There are multiple hypotheses that attempt to explain the breast cancer metastatic mechanism. However, none encompasses a “one-size-fits-all” model. One of these hypotheses, parallel progression of primary and metastatic tumors, is of particularly interest to us. It hypothesizes that, starting at initial stages of tumorigenesis, a subpopulation of the “metastatic founder cells” in the primary site of tumor are intrinsically hard-wired with capacities for future metastasis [33, 34]. In this study, we provide direct evidence to support this concept and demonstrate that AF1q-positive tumor cells in the primary site may constitute a population of the “metastatic founder cells” in breast cancer. This is supported by multi-layers of hierarchical evidence from this study. First, AF1q-enforced breast cancer cells display more aggressive, stem-like, and anoikis/drug-resistant phenotypes (Figure 1; Supplementary S2). Second, NOD/SCID models reveal that AF1q-enforced MDA-MB-231LN/AF1q cells have increased metastatic capacity as mirrored by early development of hepatic metastasis and promoting lung colonization (Figure 5). Third, AF1q-positive breast cancer cells are dynamically and intensely concentrated in the metastatic sites compared to their matched primary tissue from the same patients (Figure 6A). Fourth, we are able to confirm these *in vitro* and *in vivo* observations in a cohort of breast cancer patients in whom a higher percentage of AF1-positive breast cancer cells in primary sites were associated with significantly poorer OS, DFS and b-MFS (Figure 6B). Altogether, these coherent observations suggest that AF1q-positive cancer cells may be the “metastatic founder cells” residing in primary sites.

We also show for the first time that AF1q is a novel co-factor that directly interacts with TCF7 and forms a transcriptional complex with LEF1 and β-catenin in the Wnt signaling pathway (Figure 2A and 2F). Thus the enhancement or reduction of the TOP/FOP reporter assay results were moderate compared to changes usually seen in transcription factors. However, these results support the notion that AF1q is a co-factor through modification of the promoter binding affinity of transcriptional complex of TCF7/LEF1/Δ-catenin, which promotes higher transcriptional rates in cancer cells. Although we have shown that active AF1q binding to TCF7 is shifted into the nuclear compartment of cultured cells (Figure 2G), we were unable to demonstrate the classical nuclear signaling pattern of AF1q via IHC staining on primary tissue. This may be due to the fact that the highly acidic AF1q peptide used for raising the monoclonal antibody contains the interaction domain with TCF7 according to the Y-2-H results. Furthermore, the 9 kDa AF1q protein was predicted to be completely enveloped by TCF7 (Figure 2B) which could mask the antibody recognition site in IHC setting. Last but not least, unexplored post-translational modifications of AF1q such as phosphorylation might contribute to its localization.

Our study has further mechanistically advanced the current knowledge of AF1q that provides some of important and fundamental explanations why hematologic and solid malignancy patients with high AF1q expression have poor clinical outcomes. Patients with high AF1q expression malignancies are likely to have AF1q-driven, autonomously activated the downstream targets of the Wnt signaling pathway such as CD44 and KISS. The cellular membrane protein CD44 is an important cancer stem cell (CSC) marker and plays a critical role in many cancers' distant metastatic potential as well as AML stem cell homing to the bone marrow niche [35–37]. We reveal that AF1q is a novel regulator of CD44 expression that advances our current knowledge about the fine tuning of the Wnt signaling pathway. This work also provides a mechanistic connection to our previous observations and conundrum, why patients with myeloid malignancy with high AF1q expression have poorer OS and DFS [1, 2, 6–8]. This can be explained that the myeloid malignant stem cells are driven into a chemo-protective, sanctuary marrow niche, and escape from cytotoxic cell death.

In contrast to our current data, other groups show that AF1q can promote apoptosis through NF-kappaB mediated BAD and reactive oxygen species (ROS)-dependent signaling [3, 4, 14]. However, these observations were made in different experimental systems, such as ovarian and hepatic cancer cell lines etc., by different experimental approaches mostly *in vitro* settings. One possible explanation for this discrepancy would be that AF1q expression level has no significant difference between normal and cancerous ovarian and hepatic cell, which suggests that the biologic and oncogenic function of AF1q in these cancer cells are different compared to breast and hematologic malignancies.

AF1q synergistically activates or suppresses two groups of Wnt downstream targets that collectively create coherent driving forces for breast cancer metastasis based on RNA-seq data (Figure 4). These data further demonstrate a broad and potentially important role of AF1q in cancer stem cell and metastatic biology. Our previous and current works pivotally connect two critical regulatory axes, GATA3/miR29b and miR29b/AF1q/TCF7/CD44/KISS1, associated with the Wnt pathway for breast cancer metastasis [2, 9, 15]. Hence we provide a novel mechanistic basis for understanding AF1q's role in cellular adhesion, migration, and invasion.

This study creates an important clinical paradox that commonly used chemotherapy agents for treatment of breast or other cancer types such as doxorubicin or etoposide might potentially be “harmful” for a subgroup of breast cancer patients with an intrinsically hyperactive AF1q/CD44 regulatory axis because these chemotherapy agents can further activate the intrinsic AF1q expression and promote distant metastasis (Figure 1D). Furthermore, AF1q activation may render cancer cells more metastatic. These findings warrant more careful scrutiny of the safety and efficacy of our current neo-adjuvant and adjuvant therapeutic paradigms for breast cancer. Based on the intrinsic metastatic potential of the GATA3/miR29b/AF1q/CD44/KISS1 axes [38], these factors might need to be included in the diagnostic portfolio of the primary tumor at diagnosis. Therefore, targeting AF1q/TCF7/CD44 regulatory axis and its associated signaling molecules of the Wnt pathway may be a useful therapeutic strategy for breast cancer patients.

## MATERIALS AND METHODS

Detailed descriptions of the material and methods are presented in the supplementary data.

### Cell lines

HMLE was generously provided by Dr. Robert Weinberg (The Whitehead Institute, MIT). MCF10a, MDA-MB-231, MDA-MB-435, MDA-MB-468, HCC1143, ZR-75-1, MCF7 and Hs578T were purchased from American Type Culture Collection (ATCC). MDA-MB-231-luc-D3H2LN (MDA-MB-231LN) was purchased from Caliper Life Science.

### Transwell migration and mammosphere assay

The effect of AF1q on motility and invasiveness was determined using 8-μm pore size 24-well transwell migration chambers (Corning) and matrigel invasion chambers (BD Biosciences) as described previously [39]. For the mammosphere assay, 1% (w/v) methylcellulose was added to each complete growth medium [40]. Each sample was assayed in triplicate.

### Western blot and cellular fraction

Total cell lysates were prepared using a GLB buffer (2% SDS, 10% glycerol and 50 mM Tris, pH 6.8). Cytosolic and nuclear protein extracts were isolated using the NE-PER™ Nuclear and Cytoplasmic Extraction Kit (Thermo Scientific) according to the manufacturer's instructions. Cell lysates separated by SDS-PAGE were probed with the antibody.

### Co-immunoprecipitation (Co-IP) assays

Co-immunoprecipitation (Co-IP) assays were performed using a nuclear complex Co-IP kit (Active Motif) and analyzed by Western blot.

### Wnt pathway activity and CD44 promoter luciferase assay

To demonstrate Wnt pathway or CD44 promoter activity, the cells were transfected by the TOP/FOP reporter system (Milipore) or pGL4-CD44 promoter (−908/−118) fragment. *Renilla* plasmid (Promega) was used as an internal control.

### Electrophoretic mobility shift assay

Nuclear material was extracted from cultured cells using nuclear extraction buffer (Active Motif). EMSA was performed using the EMSA kit (Signosis) according to the manufacturer's instructions and previously described [41, 42].

### RNA-seq and IPA analysis

RNA-seq was performed to profile mRNA expression levels in enforced or suppressed expression from both AF1q enforced or suppressed MDA-MB-231LN cells in duplicate. Sequences produced by Illumina (mRNA-seq) were aligned to the human reference sequence using the BWA1, algorithm with a tolerance of 3 mismatches. Then differential transcripts deregulated more than 1.5-fold with a false discovery value of *q* < 0.05 and *P* < 0.05 were evaluated using the spring 2013 version of IPA (Ingenuity^®^ Systems, www.ingenuity.com).

### Tumor growth assay

We carried out xenograft transplants to measure tumorigenicity and metastasis *in vivo* according to approved protocol by West Virginia University IACUC committee. MDA-MB-231LN cells were transduced by the following lenti viral constructs: empty vector, enforced AF1q, shRNA control (Scr) and shRNA, respectively. Mice were imaged weekly using the IVIS imaging system for 4 weeks. Mice were euthanized and autopsied after 4 weeks post-cell injection. Mice were fed 200 mg/kg of doxycycline daily.

### Immunohistochemistry

Immunohistochemistry was performed as previously described [43]. Quantitative analysis of the staining was performed using HistoQuestTM software (TissueGnostics GmbH; www.tissuegnostics.com; Austria).

### Study population

Tissue microarrays (TMAs) from 63 female breast cancer patients who underwent surgery at the Medical University of Vienna in 1988-1994 were analyzed retrospectively under protocols approved by the institutional review board of the Medical University of Vienna [44, 45]. Each tumor was represented by triplicate core biopsies on these tissue arrays a Ret score was evaluable for all cases. Clinical and histopathological characteristics of the study population are shown in Supplementary Table S4.

### Statistical analysis

Experimental results were summarized using mean ± s.d. Student's *t*-test to assess the difference of a continuous outcome between two groups. We used Kaplan-Meier method to examine the survival curves using the statistical package of R, an open-source language and environment for statistical computing [46]. Corresponding p-values were calculated by log-rank tests as described by Harrington and Fleming [47]. Results were considered statistically significant if *P* < 0.05.

## SUPPLEMENTARY FIGURES AND TABLES


