# Supplementary material for: AF1q is a novel TCF7 co-factor which activates CD44 and promotes breast cancer metastasis
Source: Oncotarget. 2015 Jun 7;6(24):20697–710. doi: 10.18632/oncotarget.4136 (PMC4653036; doi:10.18632/oncotarget.4136)
Supplement: Supplementary file 1 [file oncotarget-06-20697-s001.pdf]

AF1q is a novel TCF7 co-factor which activates CD44 and promotes breast cancer metastasis

Supplementary Material

Cell lines

HMLE was generously provided by Dr. Robert Weinberg (The Whitehead Institute, MIT). MCF10a, MDA-MB-231, MDA-MB-435, MDA-MB-468, HCC1143, ZR-75-1, MCF7 and Hs578T were purchased from American Type Culture Collection (ATCC). MDA-MB-231-luc-D3H2LN (MDA-MB-231LN) was purchased from Caliper Life Science. Maintenance of the immortalized HMLE and MCF10a human breast epithelial cells was performed as previously described (1,2). The cells were cultured in DMEM/F-12 (1:1) medium (Invitrogen) supplemented with 5% horse serum (Gibco), 0.5 ug/ml hydrocortisone (Sigma-Aldrich), 10 ug/ml insulin (Sigma-Aldrich), and 20 ng/ml epidermal growth factor (Sigma-Aldrich). For MCF10a cells, 0.1 ug/ml cholera endotoxin (Sigma-Aldrich) was also included. The breast cancer cells were cultured in DMEM medium (Invitrogen) supplemented with 10% fetal bovine serum (Gibco). MDA-MB-231 is a metastatic line derived from spontaneous lymph node metastases of MDA-MB-231-luc breast cells.

Plasmid construction

Full-length AF1q cDNA was inserted into pLUTdNB which is pTRIPZ base modified doxycycline-inducible vector by cloning the HindIII and XhoI fragments as previously described (3). Short hairpin RNA (shRNA) of AF1q and CD44 were purchased from Open Biosystems. Empty pLUTdNB, pTRIPZ-scramble and pGIPZ-scramble (Open Biosystem) were used as controls for pLUTdNB-AF1q, pTRIPZ-AF1q shRNA, and pGIPZ-CD44 shRNA, respectively.

Viral production and infection

Lentivirus was produced by co-transfection of HEK 293T cells (ATCC) with the lentiviral constructs pVSV-G and psPAX2 (Addgene). Transfections were carried out using Lipofectamine 2000 (Invitrogen). For enforced AF1q expression or shRNA targeting, cells underwent lentiviral transduction and were selected using 1 μg/ml puromycin (Thermo Fisher) as previously described (4). After antibiotic selection, cells were cultured in complete medium including 1 μg/ml doxycycline (Thermo Fisher) for AF1q or shRNA induction.

Chemo-resistance Assay

Cell cytotoxicity was assessed by measuring the reducing power of live cells. Cells were seeded and then incubated with 100 nM of Doxorubicin (MP Bio) or 100 μM of Etoposide (MP Bio) at 37°C. After 24h, cells were washed with PBS to remove dead cells. Reducing power was assayed using the Prestoblue (Invitrogen) according to the manufacturer's instructions. Experiments were performed in triplicates with three repeats for each experiment.

Transwell migration assay

The lower side chambers were filled with 750 μL of complete medium. Cells (2.5×10^3 cells per insert for migration and HMLE 2.5×10^5 and others 2.5×10^4 cells per insert for invasion) were seeded in the upper side chambers with basal medium with 0.1% BSA (MP Bio). Cells were allowed to migrate 16 h for the migration assay and 24 h for the invasion assay. Non-migrated cells were removed with cotton swabs, and migrated cells were fixed with ice-cold methanol and stained with Kwikdiff stain kit (Thermo Scientific). Images were captured using the camera on a Zeiss inverted microscope with magnification x100. Cells that migrated to the lower side of the filter were quantified by manual counting.

Mammosphere assay

Cells were trypsinized to obtain a single cell suspension, and then diluted to a concentration of 1×10^4 cells/ml for HMLE or 2×10^4 cells/ml for other cells in Opti-MEM (Invitrogen). 1 ml of 1% (w/v) methylcellulose medium was added per well to 24-well ultra-low attachment surface plates (Costar). 200 µl of prepared cell suspension was seeded into each well containing 1% (w/v) methylcellulose medium and mixed vigorously. Media was replenished every 3-4 days for 10-21 days.

RNA and miRNA extraction and RT-qPCR analysis

The total RNA extracted with *mir*Vana miRNA isolation kit (Invitrogen) was reverse transcribed using High Capacity cDNA Reverse Transcription kits (Applied Biosystems) according to the manufacturer's instructions. qPCR was performed on an ABI 7500 System (Applied Biosystems), using TaqMan Universal PCR Master Mix (Applied Biosystems). The following sense (S) and antisense (AS) primers were used to amplify CD44 by qPCR; S: 5'-TCCAACACCTCCCAGTATGACA-3', AS: 5'-GGCAGGTCTGTGACTGATGTACA-3'. miRNAs were detected by RT-qPCR using the TaqMan miRNA Assay. mRNA and miRNA29b expression was normalized to HPRT1 and RNU48, respectively. All samples were run in triplicate and mean value and s.d. were calculated.

Western blot and Cellular fraction

For blots of whole-cell lysates, cells were lysed directly in GLB buffer (2% SDS, 10% glycerol and 50 mM Tris, pH 6.8), boiled, and separated by electrophoresis on a 4 – 12% SDS-PAGE gradient gel. Proteins were transferred to PVDF membrane (Millipore) and blocked in 5% skim milk (Bio-Rad) in 0.05% PBST. Rabbit monoclonal anti-AF1q antibody was co-developed with Epitomics. Anti-CD44, TCF7 and β-actin monoclonal antibodies were purchased from Cell

Signaling Technology, Inc. After the appropriate antibody incubations, an enhanced chemiluminescence (Denville) system was used for developing blots.

Cellular fractionation was performed using the NE-PER™ Nuclear and Cytoplasmic Extraction Kit (Thermo Scientific) according to the manufacturer's instructions. Protein concentration was evaluated in each fraction using a BCA kit (Thermo Scientific) after isolation and then performed Western blotting as described above using AF1q antibody. GAPDH and Histone H3 (Santa Cruz) were used for internal loading control.

Anoikis Assay

Cells were dissociated and plated (2×10^5 cells/well) into a 6-well Ultra-Low Attachment Cluster Dish (Costar) with normal growth media for 24 hours to induce anoikis. Apoptosis was analysed using Dead Cell Apoptosis Kit with Annexin V APC and SYTOX Green (Invitrogen). Apototic cells and dead cells were detected using annexin V expression and SYTOX nucleic acid staining by flow cytometry, respectively. All samples were analyzed triplicate and cells plated normal culture plate were used as a control.

Yeast-2-Hybrid screening

Yeast 2-hybrid screening was performed using the ULTImate Y2HTM system (Hybrigenics Services). Recombination with the N-LexA-bait-C fusion vector pB27 plasmid and AF1q (1-90) generated the yeast bait constructs. The library construction of human thymocytes (CD4+, CD8+) RP1 subcloned into pB27 was screened following the manufacturer's protocols.

Co-immunoprecipitation (Co-IP) assays

MCF10a cells were fractionated and nuclear fractions were incubated with anti-TCF7 antibody on a rocker at 4°C overnight. Immunocomplexes were pulled down by incubating with Protein A-Sepharose (Cell Signaling) for 1 h at 4°C, followed by washing 3 times with ice-cold

lysis buffer to eliminate non-specific interactions. Protein A-sepharose-bound immunocomplexes were then resuspended in SDS-PAGE loading buffer, boiled for 10 min, and analyzed by Western blot as described above.

Wnt pathway activity assay

Dual-Luciferase reporter assays were performed using a TOP/FOP reporter system (Milipore). Briefly, 1×10^4 cells were plated in each well of a 96-well plate. The next day, the reporter plasmid (100 ng) and *Renilla* plasmid (10 ng, Promega) were co-transfected into the cells with lipofectamine 2000 (Invitrogen). After 48 hours, cells were washed with PBS and assayed with the Dual-Luciferase reporter assay system (Promega) according to the manufacturer's instructions. Luciferase activity was determined using EnVision 2104 Multilabel reader (PerkinElmer). Quantitation of luminescent signal from reporter plasmid was normalized by quantitation of the luminescent signal from *Renilla*.

CD44 promoter luciferase assay

To construct reporter plasmids, 890 bases of a CD44 (-908/-118) promoter fragment were cloned to between XhoI and HindIII sites of the pGL4-Luciferase reporter plasmid (Promega) (5). Luciferase activity was measured as described above.

Electrophoretic mobility shift assay

The reaction mixtures (10 μl) containing 5 μg of nuclear extract, 1X binding buffer, 50 ng of poly (dI-dC) and 20 ng of Alexa 488-labeled CD44 promoter region probe (5'–CTTTTCCTTTGAGGCCTGCCTCAA-3', IDT) incubate for 30 min at room temperature. Reaction mixtures containing no nuclear extract were also incubated with labeled probes as negative control samples. Each sample was loaded onto a 6% polyacrylamide gel in 0.5X

Tris/Borate/EDTA (TBE) buffer. For the antibody super-shift analysis, 1 μg of antibody was added to the nuclear extracts prior to incubation with the Alexa-488 labeled probe for 30 min. In competition analysis, 100-fold molar excess of the unlabeled double-strand oligonucleotide was added to the reaction mixture prior to the addition of the labeled probe. Samples were electrophoresed until the dye had reached 1 inch from the bottom of the gel and then scanned with the Typhoon 9410 imager (GE Healthcare).

RNA-seq

We used Cuffdiff 24 to perform differential expression testing to detect the differential expressed genes. Genes with q<0.05 were defined to be statistically differentially expressed. Finally, the differentially expressed genes identified were used for gene set enrichment analysis with GSEA25 and pathway enrichment analysis with DAVID Bioinformatics resources 26. Gene lists of KEGG pathway signatures and stem cell signatures were obtained from GSEA MSigDB website (http://www.broadinstitute.org/gsea/msigdb /index.jsp) for GSEA analysis in our cohort of mRNA expression data.

Transcriptome and Interactive pathway analysis

RNA was isolated from both AF1q-enforced and shRNA-transduced MDA-MB-231 cells and sequenced by the CCHMC Genetic Variation and Gene Discovery Core Facility (http://dna.chmcc.org/www/nextgen_main.php). Differential transcripts deregulated more than 1.5-fold with a false discovery value of q<0.05 and P<0.05 were evaluated. To uncover significantly enriched and significantly deregulated pathways in that gene set, the data were analyzed using the spring 2013 version of IPA (Ingenuity® Systems, www.ingenuity.com).

Tumor growth assay

The MDA-MB-231 transduced with various lenti viral constructs were accordingly injected into 6-week old NOD/SCID-gamma C-/- immune-deficient female mice (WVU). Animals were divided into four groups, control, AF1q, Scr and shRNA and each group includes 5 mice. The cells were diluted with PBS to a concentration of 1×10^7 and 100 µl of cell suspension was injected into the abdominal mammary fat pad in female mice (1×10^6 cells). To measure colonization ability, MDA-MB-231 or MDA-MB-231LN/AF1q cells (1×10^5 cells) were injected into the tail-vein in 6-week old NOD/SCID-gamma C-/- immune-deficient male mice (n=3). Mice were imaged weekly using the IVIS imaging system for 4 weeks. Mice were euthanized and autopsied after 4 weeks post-cell injection. Mice were fed 200 mg/kg of doxycycline daily.

Immunohistochemistry

Tissue samples were fixed in 4% paraformaldehyde, embedded in paraffin, and then 4-µm sections were prepared. Sections were de-waxed and a steamer pre-treatment in Tris/EDTA buffer (DAKO) was performed. Endogenous peroxidase activity was quenched by incubation in 3% hydrogen peroxide in PBS. For blocking steps, avidin (Sigma-Aldrich), biotin (Sigma-Aldrich) in PBS, and a super block (IDlabs Biotechnology) were used. Rabbit monoclonal AF1q antibody in a 1:200 dilution was incubated at 4°C overnight. IHC detection was performed with the IDetect Super Stain System HRP (IDlabs Biotechnology). Specific signals were amplified using 3-amino-9-ethylcarbazole (IDlabs Biotechnology) under visual control, followed by counterstaining with hematoxylin.

1. Mani SA, Guo W, Liao MJ, Eaton EN, Ayyanan A, Zhou AY, et al. The epithelial-mesenchymal transition generates cells with properties of stem cells. Cell 2008;133(4):704-15.
2. Debnath J, Muthuswamy SK, Brugge JS. Morphogenesis and oncogenesis of MCF-10A mammary epithelial acini grown in three-dimensional basement membrane cultures. Methods 2003;30(3):256-68.
3. McLaughlin SL, Ice RJ, Rajulapati A, Kozyulina PY, Livengood RH, Kozyreva VK, et al. NEDD9 depletion leads to MMP14 inactivation by TIMP2 and prevents invasion and metastasis. Molecular cancer research : MCR 2014;12(1):69-81.
4. Park JO, Park SH, Hong ST. A new method for transduction of mesenchymal stem cells using mechanical agitation. Molecules and cells 2009;28(6):515-20.
5. Muller I, Wischnewski F, Pantel K, Schwarzenbach H. Promoter- and cell-specific epigenetic regulation of CD44, Cyclin D2, GLIPR1 and PTEN by methyl-CpG binding proteins and histone modifications. BMC cancer 2010;10:297.

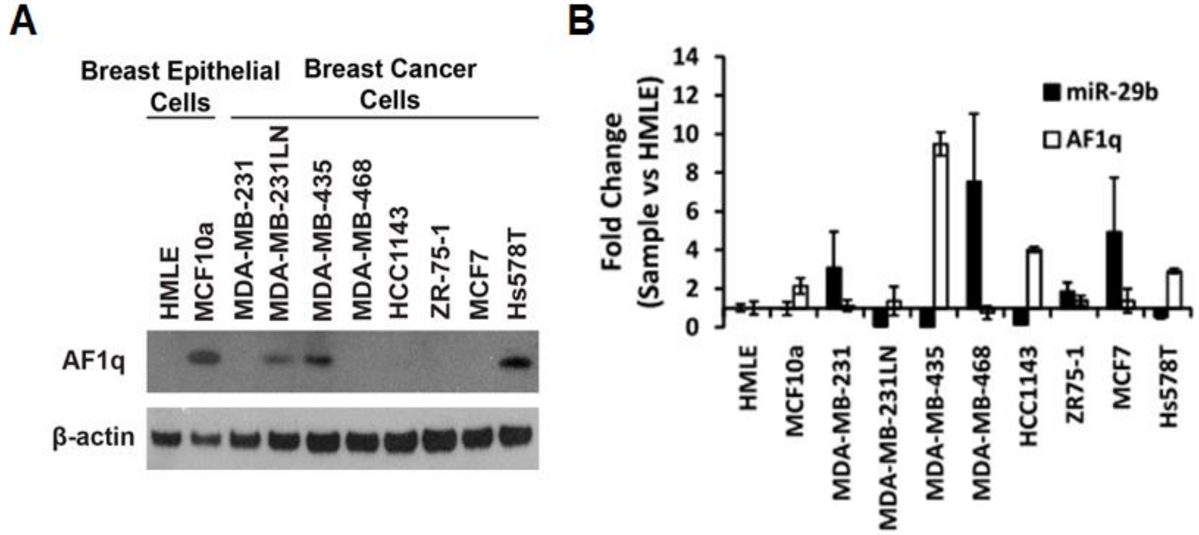

Supplementary Figure S1: The AF1q expression status in breast epithelial and cancerous cell lines was inversely related to miR29b expression status. (A) AF1q expression status in breast normal and cancer cell lines. **(B)** miR29b expression was validated by TaqMan qPCR. These results were consistent with our finding in leukemia that miR29b regulates AF1q expression in these breast epithelial and cancerous cell lines. Data were summarized with mean values ± s.d. of three independent experiments.

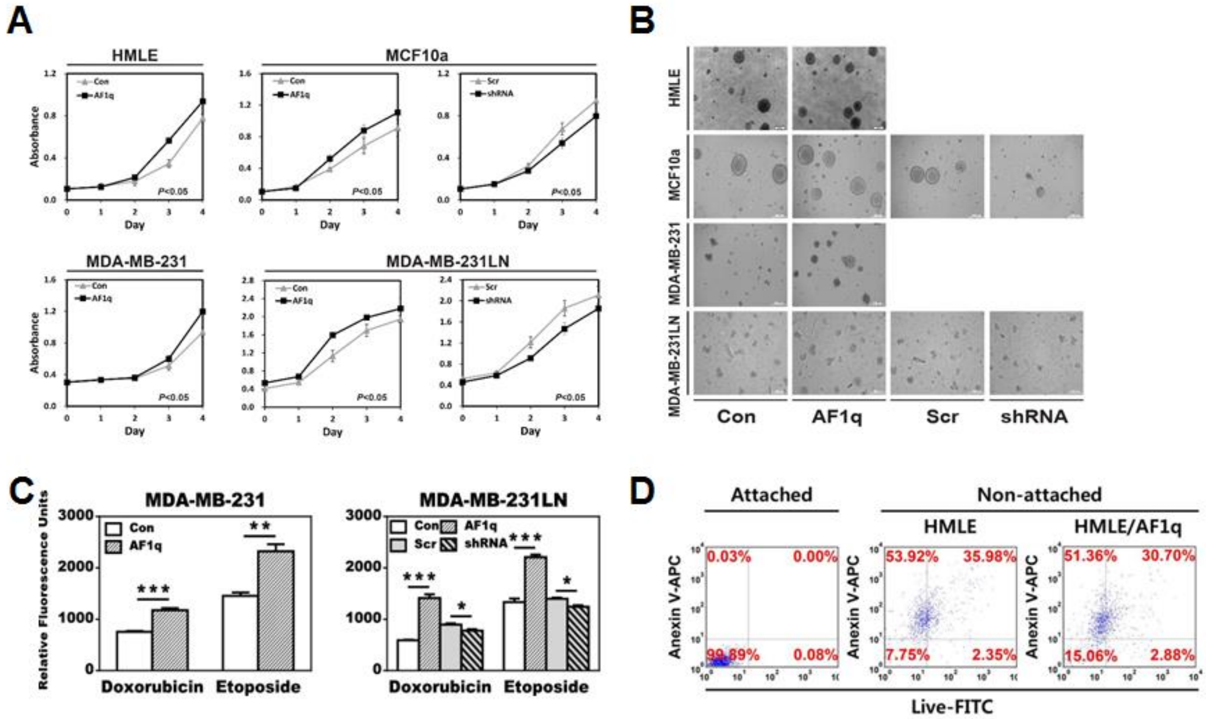

Supplementary Figure S2: The role of AF1q in breast cancer metastasis. (A) Enforced AF1q expression in normal (HMLE and MCF10a) and cancerous (MDA-MB-231 and MDA-MB-231LN) breast cells promoted a significant cell growth. Data were summarized with mean values ± s.d. of three independent experiments. **(B)** Enforced AF1q expression in breast epithelial cells (HMLE and MCF10a) or breast cancer cells (MDA-MB-231 and MDA-MB-231LN) enhanced mammosphere or tumorsphere formation, and suppressed endogenous AF1q expression in MCF10a or MDA-MB-231LN cells attenuated mammosphere or tumorsphere formation. **(C)** In a chemo-resistant assay, enforced AF1q expression in MDA-MB-231 and MDA-MB-231LN inhibits doxorubicin- and etoposide-induced apoptosis compared to their correspondent controls; Suppressed the endogenous AF1q expression with shRNA sensitizes. Data were summarized with mean values ± s.d. of three independent experiments. *P* values were calculated using student *t* test (*, $P<0.05$; **, $P<0.01$; ***, $P<0.001$). **(D)** Enforced AF1q expression in breast epithelial HMLE cells produced a side population of cells that were resistant to anoikis.

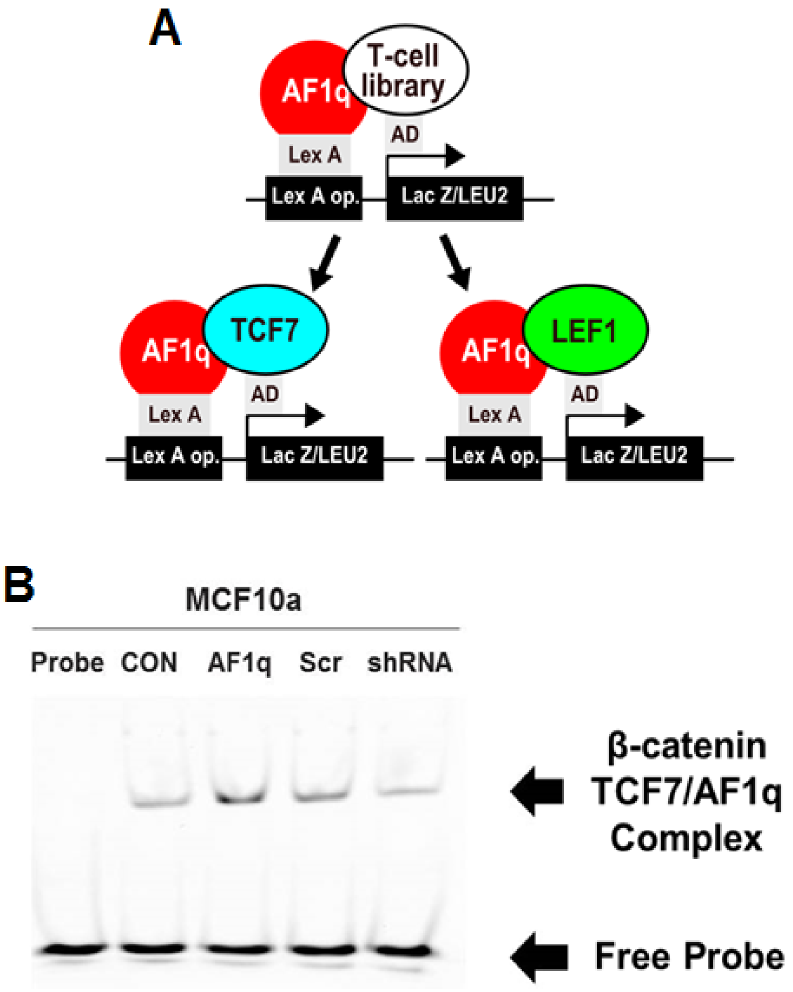

Supplementary Figure S3: Y-2-H screens suggested that AF1q had a high affinity for TCF7 and possibly for LEF1. **(A)** Illustration of the possible interaction between AF1q and TCF7 or LEF1 by Y-2-H. **(B)** EMSA analysis, performed with a CD44 promoter probe with a TCF7/LEF1 binding motif, demonstrated that enforced AF1q expression promoted its binding to the CD44 promoter, but suppressed endogenous AF1q expression attenuated its binding to the CD44 promoter.

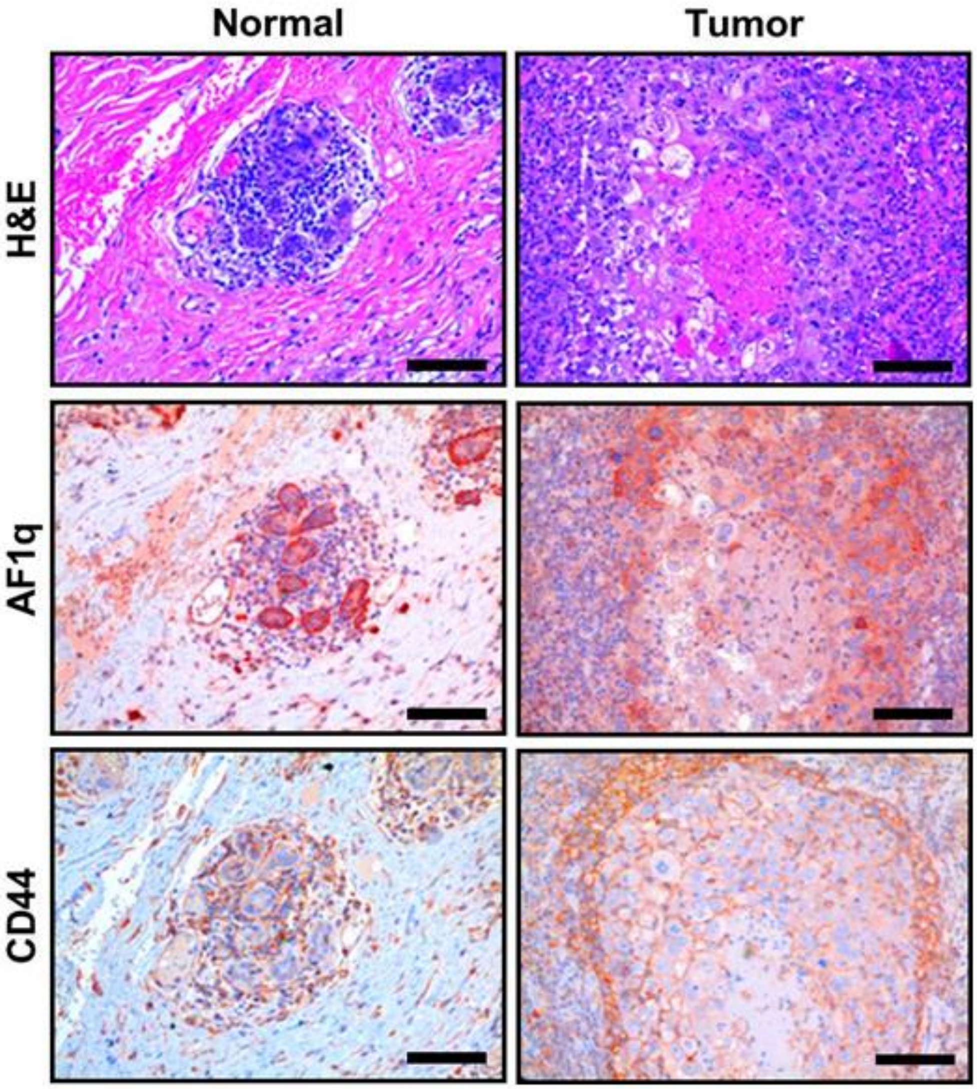

Supplementary Figure S4: AF1q and CD44 IHC showed that AF1q expression areas matched with CD44 signals in breast normal and tumor tissues. Scale bars = 50 µm.

Supplementary Table S1. Interactive partner fragment analysis.

AF1q Interactor	Start/Stop nt	Stop Codon
TCF7	324-867	X
TCF7	344-783	
TCF7	348-976	X
TCF7	363-866	X
TCF7	375-971	X
TCF7	375-971	X
TCF7	375-971	X
TCF7	421-910	X
LEF1	564-1134	X
LEF1	582-1153	X

Supplementary Table S2. The genes associated with enforced or suppressed AF1q expression in MDA231LN cells based on RNA-seq.

Gene	Locus	log2 fold change.	*p*_value	*q*_value
ATP8B1	chr18:55313657-55405276	-0,615024	0,000178165	0,00780973
SCD	chr10:102106876-102124591	-1,49123	0	0
PLAT	chr8:42032235-42065242	0,650568	7,9e-09	8,84e-07
SERPINE1	chr7:100770369-100782547	0,663831	1,59e-05	0,000950716
AREG	chr4:75310850-75320726	1,09122	7,95e-10	0.0000001
IFIT3	chr10:90973325-91174314	1,24658	0	0
SLPI	chr20:43880879-43883205	1,63766	0,00040172	0,0153122
NR1D1	chr17:38219062-38256978	1,20883	6,79e-09	7,64e-07
KRT17	chr17:39775688-39781094	1,26294	5,38e-06	0,000358676
GDF15	chr19:18451396-18665861	3,34089	0	0
TNS4	chr17:38632079-38657854	2,53811	0,000906959	0,0298562
OASL	chr12:121458120-121477045	1,03511	1,21e-10	1,71e-08
KLF4	chr9:110247132-110252763	1,30225	1,08e-12	1,91e-10
STRA6	chr15:74471806-74501371	1,52229	1,51e-05	0,000909146
IFI44	chr1:79034379-79129763	0,786835	0,000228695	0,00966844
DUSP5	chr10:112257595-112271302	0,726658	9,24e-07	7,36e-05
ADAMTS14	chr10:72432558-72522197	1,15877	4,9e-07	4,07e-05
PMAIP1	chr18:57567179-57571538	1,11131	1,15e-10	1,64e-08
PTGES	chr9:132500609-132515326	0,64354	6,66e-14	1,35e-11
ANXA8L1	chr10:47011752-47174122	1,4097	0	0
DHRS3	chr1:12627938-12677737	1,31636	5,68e-08	5,57e-06
PTX3	chr3:156893011-157251408	-0,683528	2,06e-06	0,000151192
IFI27	chr14:94577078-94583033	1,33485	0	0
C15orf48	chr15:45694528-45848928	0,920217	4,42e-08	4,39e-06
GPRC5B	chr16:19717675-19896239	0,731378	0,000229551	0,00968007
IGFBP6	chr12:53491435-53496128	0,925279	4,24e-08	4,23e-06
SOST	chr17:41831102-41836156	1,1615	1,42e-06	0,000108971
DDIT4	chr10:74033677-74035794	1,3292	2,22e-15	5,07e-13
SDPR	chr2:192699027-193060435	-1,18243	7,52e-13	1,38e-10
KISS1	chr1:204159468-204165628	-1,30567	1,02e-12	1,83e-10
ALDH1A3	chr15:101419580-101610317	0,76771	8,64e-06	0,000555728
INSIG1	chr7:155089485-155101945	-0,803265	0,000528239	0,0192115
ISG15	chr1:948802-949920	0,637395	2,93e-13	5,88e-11
HLA-DRB1	chr6:32546545-32578053	1,42032	5,36e-06	0,000358676
PLCG2	chr16:81812906-81991898	0,760984	0,000495007	0,0182823
HLA-C	chr6:31236525-31239907	0,626022	6,66e-15	1,48e-12
HLA-F	chr6:29690551-29716826	1,18087	0,000106887	0,00513087
SAMD9	chr7:92728828-92747336	0,971292	1,48e-06	0,000113226
HLA-A	chr6:29867167-29913661	0,677834	0	0
MLLT11	chr1:151020215-151042801	7,63763	0	0
RN28S1	chrX:108297360-108297792	-1,33425	0	0
HLA-B	chr6:31321648-31325406	0,835747	0	0
AC079949.1	chr12:127650452-127650987	-1,12765	0	0
AC116340.1	chr5:71146739-71146942	-1,19811	0	0
AC006368.1	chr2:229888689-230136001	-1,31738	0	0
AP003035.1	chr11:83166054-85338966	-1,61491	0	0
AC108078.1	chr4:70296578-70296753	-1,45133	0	0
CFB	chr6:31865561-31919861	1,84048	6,19e-10	8,05e-08
AC104837.2	chr1:79034379-79129763	0,691776	7,76e-05	0,00384644
AC098691.1	chr1:91726322-91870426	-1,15751	0	0
AC097532.1	chr2:133038646-133038738	-1,40478	4,42e-09	5,11e-07

Supplementary Table S3. The RNA-seq data set of MDA231-LN/AF1q was subjected to Ingenuity Pathway Analysis (IPA). The significance of the association on functions was measured. *p*-value is indicative of enrichment in the group. Enforced AF1q expression shows that the cell proliferation and metastasis associated clusters are the highest activated group.

Category	Functions Annotation	p-Value	Predicted Activation State	Activation z-score
Cellular Movement	invasion of cells	1,76E-14	Increased	3,301
Cell Death and Survival	cell viability	1,48E-18	Increased	3,265
Cellular Function and Maintenance	cellular homeostasis	9,70E-08	Increased	3,197
Cell Death and Survival	cell survival	4,83E-19	Increased	3,078
Cellular Movement	migration of cells	2,28E-14	Increased	2,985
Cellular Movement	cell movement	1,89E-14	Increased	2,967
Cellular Movement	invasion of tumor cell lines	1,00E-11	Increased	2,548
Cancer	cancer of cells	6,34E-07	Increased	2,502
Cancer	metastasis of cells	3,10E-06	Increased	2,502
Cardiovascular System Development and Function	angiogenesis	9,50E-10	Increased	2,400
Cell Death and Survival	cytotoxicity	4,91E-06	Increased	2,400
Cancer	cancer of tumor cell lines	3,58E-05	Increased	2,270
Cancer	metastasis of breast cancer cell lines	1,65E-05	Increased	2,219
Reproductive System Disease	metastasis of breast cancer cell lines	1,65E-05	Increased	2,219

Supplementary Table S4. Correlation of AF1q status with established clinical and histopathological parameters of human breast cancer.

	AF1q	high (n=21)		low (n=42)		p-value
Age of onset						
<50	(n=22)	8	(38.1%)	14	(33.3%)	0.709
≥50	(n=41)	13	(61.9%)	28	(66.7%)	
Tumor type						
ductal	(n=49)	21	(100%)	28	(70.0%)	0.005
lobular	(n=12)	0	(0%)	12	(30.0%)	
other, NA	(n=2)	0		2		
Tumor size						
pT1	(n=13)	3	(14.3%)	10	(24.4%)	0.355
pT2-4	(n=49)	18	(85.7%)	31	(75.6%)	
NA	(n=1)	0		1		
Tumor grade						
pG1-2	(n=33)	9	(42.9%)	24	(58.5%)	0.242
pG3	(n=29)	12	(57.1%)	17	(41.5%)	
NA	(n=1)	0		1		
Tumor stage						
1	(n=8)	1	(5.0%)	7	(18.9%)	0.149
2-4	(n=49)	19	(95.0%)	30	(81.1%)	
NA	(n=6)	1		5		
Lymph node status						
pN0	(n=17)	2	(10.5%)	15	(38.5%)	0.028
pN+	(n=41)	17	(89.5%)	24	(61.5%)	
NA	(n=5)	2		3		
ER status						
neg	(n=39)	18	(85.7%)	21	(50.0%)	0.006
pos	(n=24)	3	(14.3%)	21	(50.0%)	
PR status						
neg	(n=43)	14	(70.0%)	29	(70.7%)	0.953
pos	(n=18)	6	(30.0%)	12	(29.3%)	
NA	(n=2)	1		1		
HER2 status						
neg	(n=49)	15	(75.0)	34	(81.0)	0.590
pos	(n=13)	5	(25.0)	8	(19.0)	
NA	(n=1)	1		0		
p53 status						
neg	(n=41)	13	(61.9%)	28	(66.7%)	0.709
pos	(n=22)	8	(38.1%)	14	(33.3%)	
Ki67 pos cells						
<10%	(n=38)	14	(66.7%)	24	(61.5%)	0.694
≥10%	(n=22)	7	(33.3%)	15	(38.5%)	
NA	(n=3)	0		3		

The number of patients (n) and their relative frequencies (in %; figures in parentheses) in the indicated clinical and histophathological categories are shown. AF1q high, >50% positive tumor cells; AF1q low, ≤50% positive tumor cells; p-value, Chi-square test p-values; pN0, no lymph node metastases; pN+ patient has lymph node metastases; ER, estrogen receptor; PR, progesterone receptor; NA, status not available. Note that NA subjects were not included in the calculations of percentages and p-values.
